# Exploring association between pseudoexfoliation syndrome and ocular aging

**DOI:** 10.1007/s10792-022-02486-0

**Published:** 2022-09-21

**Authors:** Ugne Rumelaitiene, Martynas Speckauskas, Abdonas Tamosiunas, Ricardas Radisauskas, Tunde Peto, Morten Bøgelund Larsen, Dalia Zaliūniene

**Affiliations:** 1 Department of Ophthalmology, University of Health Sciences, Mickeviciaus str. 9, 44307 Kaunas, Lithuania; 2 Institute of Cardiology, Lithuanian University of Health Sciences, 50162 Kaunas, Lithuania; 3 Department of Environmental and Occupational Medicine, Lithuanian University of Health Sciences, 47181 Kaunas, Lithuania; 4 Centre for Public Health, Faculty of Medicine and Health Sciences, Queen’s University Belfast, Belfast, UK

**Keywords:** Pseudoexfoliation syndrome prevalence, Cataract, Central corneal thickness, Corneal curvature, Age-related macular degeneration

## Abstract

**Purpose:**

Within a population-based follow-up study, to examine the 10-year incidence of pseudoexfoliation syndrome (PEX), possible risk factors for PEX and its association with ocular aging of the cornea, lens and retina.

**Methods:**

The baseline examination was conducted in 2006 on a random sample of 1,033 adult participants from Kaunas city (Lithuania) population of whom 631 had ophthalmic examination data at attendance of the 10-year follow-up in 2016. Detailed examination of the anterior and posterior segment of the eye was carried out. After diagnostic mydriasis PEX was diagnosed by the presence of typical grayish-white exfoliation material on the anterior capsule surface of the lens. The participants were divided to PEX and non-PEX groups.

**Results:**

PEX prevalence increased from 9.8 to 34.2% from baseline to 10-year follow-up. Nuclear cataract was common both in the PEX group (66.7%) and in those without PEX (72.2%), but this difference did not reach statistically significantly increased risk of developing cataract in those with PEX (OR 1.2; *p* = 0.61). Central corneal thickness (CCT) was thinner in the PEX group (529 ± 34 μm) and in the oldest group (525 ± 36 μm) (*p* < 0.001). Compared to baseline, corneal curvature (CC) became flatter in both groups (7.6 ± 0.27 vs 7.7 ± 0.26 mm; *p* < 0.001) during the follow-up, but the difference did not reach significance between groups. Corneal astigmatism was most commonly with-the-rule in both groups (37 (50.0%) vs 148 (68.5%); *p* > 0.05). Age, sex and PEX had no influence on age-related macular degeneration distribution.

**Conclusion:**

The prevalence of PEX increased significantly with age in our population, with those with PEX having thinner and flatter corneae, but no difference in cataract and age-related macular degeneration characteristics.

**Supplementary Information:**

The online version contains supplementary material available at 10.1007/s10792-022-02486-0.

## Introduction

Pseudoexfoliation syndrome (PEX) is an age-related disorder in which greyish-white flakes accumulate in the anterior segment of the eye. PEX pathogenesis is not completely understood [1]. PEX fibrils appear to be produced by various unrelated endothelial, epithelial, mesenchymal cell types, including lens epithelial cells, trabecular meshwork cells, ciliary epithelial cells, smooth muscle cells and fibroblasts [2] indicating that PEX might be a part of a generalized ageing disorder [3].

Approximately 0.2–30.0% of the global population older than 60 years of age is affected by PEX [4, 5]. The highest PEX prevalence rates, as high as 40.6% in those aged 80 years or over [6], have been reported in Nordic countries [6, 7]. Clinically unilateral ocular involvement varies from 48.0 to 76.0% of patients. Progression to bilateral PEX was described in up to 50.0–71.0% of patients within 5–12 years after diagnosis [8, 9].

A possible correlation has been established between PEX and steeper corneal curvature (CC), central corneal thickness (CCT) and nuclear lens opacifications [10, 11], but not with age-related maculopathy [10].

There is no clearly established gender predilection [12] with conflicting data between Kiliç’s finding for a significant relationship between PEX and advancing age and male sex [13], while the 12-year follow-up of Reykjavik Eye Study, Iceland, found an association with older age and female sex [9]. We are yet to find any follow-up studies that examine PEX and its associations with ophthalmological changes in Baltic countries. In 2006, a population-based epidemiological study that was part of larger Health, Alcohol and Psychosocial Factors In Eastern Europe (HAPIEE) [14] study was conducted in the Hospital of Lithuanian University of Health Sciences. HAPPIE examined the potential associations of ophthalmological and cardiovascular diseases. After 10 years (2016), those willing and able to come back for a follow-up was re-examined.

The aim of this paper was to determine associations of PEX with ocular changes in Lithuanian urban population and identify possible risk factors of PEX 10 years after the baseline study.

## Materials and methods

### Study sample/study population

During 2006–2008, 7087 subjects from Kaunas city (Lithuania) participated in international Health, Alcohol and Psychosocial Factors in Eastern Europe (HAPIEE) study. In 2006, out of the 7087 invited, 1065 individuals participated in the ophthalmological sub-study [14]. In 2016–2017, 686 subjects were invited and examined in a 10-year follow-up study. The study was approved by the Regional Bioethics Committee and was carried out in accordance with the Declaration of Helsinki. During the study, informed consent was obtained from each participant.

At HAPPIE baseline visit in 2006, ophthalmological examination was carried out on 1,033 participants. Ten years later, 686 returned for a follow-up examination (response rate of 66.4%; 239 males (37.9%) and 392 females (62.1%)). Altogether, 347 individuals did not return due to death (*n* = 164) and migration/refusal (*n* = 183).

Of the 686 examined, 55 respondents were not included in the current data analysis as their PEX status could not be determined due to trauma/phthisis, pseudophakia or aphakia in both eyes and lens subluxation, resulting in a cohort of 631 to be included in this study.

Weight and height were measured with a calibrated medical scale and without shoes or heavy clothes. Body mass index (BMI) was calculated as the weight in kilograms divided by the height in meters squared (kg/m^2^). Normal weight was defined as BMI < 25.0 kg/m^2^, overweight as BMI ≥ 25.0–29.99 kg/m^2^ and obesity as BMI ≥ 30.0 kg/m^2^ [15].

### Study instrument

All participants underwent ophthalmological examination according to a standard examination protocol and same methodology as at baseline [16–18]. The examination was carried out by two trained and certified ophthalmologists who had no access to the subjects’ medical history.

PEX was diagnosed by slit-lamp examination after diagnostic mydriasis with 1 drop of 1% cyclopentolate. PEX was confirmed as definite by the presence of typical grayish-white exfoliation material on the anterior capsule surface of the lens (complete or partial peripheral band and⁄ or a central shield). Other changes associated with PEX such as grayish-white deposits elsewhere in the anterior chamber (iris, cornea), precapsular frosting or haze, supported the diagnosis of PEX. PEX was deemed suspect/possible if precapsular frosting or haze was seen. The participants were classified as having PEX if any typical pseudoexfoliation material was present in at least one eye. For statistical analysis, we used data of the respondents with definite PEX diagnosis as the PEX group. Persons with suspected PEX were grouped together with those without any signs of the PEX.

All study respondents answered a standard questionnaire regarding lifestyle, subjective health and ophthalmological pathology (supplement 1).

### Ophthalmological examination

For the 10-year follow-up study, 1262 eyes of 631 individuals' ophthalmic examination results were included. Lens opacification was evaluated at slit lamp by LOCS III international classification: NO/NC—nuclear opalescence/color (evaluation 0.1 to 6.9), C—cortical, P—posterior subcapsular (evaluation 0.1–5.9) [19]. Cataract was evaluated in 1262 eyes.

After diagnostic mydriasis as above, fundus photographs centered on the fovea were taken using Canon CF-60Uvi (Canon Medical Systems, USA). Retinal images were graded by trained and certified ophthalmic graders at Moorfields Eye Hospital Reading Centre in London, UK, primarily based on the International Classification for age-related macular degeneration (AMD). The grading of photographs was carried out by the same graders for both baseline and follow-up studies data [20, 21]. Altogether, 1262 eyes had retina images graded; of these, 45 (3.6%) in baseline and 65 (5.2%) in follow-up had ungradable images due to cataract.

Central corneal pachymetry was measured with pachymeter (ALCON OCUSCAN RxP, Alcon Laboratories inc., USA), in auto mode, averaging 10 readings. In the 10-year follow-up, CCT was measured in 1260 eyes, with only 2 eyes' measurement missing. In the baseline study, eyes were selected randomly for this examination and so only 304 eyes were available for comparison.

Keratometry was taken using the Auto keratorefractometer (ACCUREF-K 9001, SHIN–NIPPON Commerce, inc., Japan). K1, K2, K1-axis, K2-axis and corneal curvature measurements were recorded from keratometry. Corneal astigmatism was calculated as follows: K1–K2 = corneal astigmatism (cylinder (diopters (D)). If the cylinder was < 1.0D, it was considered as no corneal astigmatism; if it was ≥ 1.0D, it was considered as corneal astigmatism. Corneal astigmatism was classified as with-the-rule (ax 90 ͦ ± 30 ͦ), against-the-rule (ax 0 ͦ ± 30 ͦ) and oblique (all the left). The corneal curvature (CC) results were documented from auto keratorefractometer. CC and astigmatism were recorded reliably in 1182 eyes; in 80 eyes dry eye prevented reliable measurements to be taken.

For cataract, AMD, CCT, CC, corneal astigmatism measurements each eye was kept in the analysis. For the incidence of PEX the same eye of the same subjects was evaluated.

### Statistical analysis

Statistical analysis was performed using IBM SPSS Statistics version 20 software. Descriptive statistics were applied for various data points in the PEX and non-PEX groups. Unilateral and bilateral PEX cases were separated into subgroups. Quantitative variables were presented as median, and interquartile range or mean and standard deviation (SD). Categorical data were presented as number (percent).

Data normality detection of continuous variables was checked using *Kolmogorov–Smirnov test*. In case of non-normality, medians and interquartile ranges (IQR) were calculated and Mann–Whitney *U*-test was used to compare continuous data between groups. *Chi-square (χ*^2^) test or *Fisher exact 2-sided test* was used to compare categorical variables. For ordinal data *χ*^2^* linear-by-linear* association test was used for confirmation of the linear trend. The comparison of proportions between groups was performed using *z test*. *McNemar's χ*^2^* tes*t was used to assess the difference between paired proportions. Quantitative variables were compared with *Wilcoxon test.*

Binary logistic regression analyses were conducted with PEX as predictor controlling for age and gender. Odds ratios (OR) and 95% confidence intervals (CI) of OR were calculated for the risk of new PEX cases and the dependence of risk factors.

## Results

Altogether, 631 participants had full examination (392 (62.1%) male and 239 (37.9%) female). Baseline and follow-up characteristics are presented in Tables 1 and 2. The prevalence of PEX in the baseline cohort (2006–2008) was 9.8% (Table 2) [16].

**Table 1:** The distribution of pseudoexfoliation syndrome in follow-up study by sex and affected eye (unilateral, bilateral)

	Non-PEX, *n* (%)	PEX, *n* (%)	*p*-value	PEX, *n* (%)		*p*-value
				Unilateral	Bilateral	
Male	154 (64.4)	85 (35.6)	*p* > 0.05	35 (41.2)	50 (58.8)	*p* > 0.05
Female	261 (66.6)	131 (33.4)		51 (38.9)	80 (61.1)	
Total	415 (65.8)	216 (34.2)		86 (39.8)	130 (60.2)	

*Non-PEX* pseudoexfoliation syndrome not diagnosed; *PEX* pseudoexfoliation syndrome

**Table 2:** The distribution of pseudoexfoliation syndrome in baseline and follow-up studies

	Non-PEX, *n* (%)	PEX, *n* (%)	Total, *n* (%)	Unilateral PEX, *n* (%)	Bilateral PEX, *n* (%)	Total, *n* (%)	*p*-value
Baseline study	569 (90.2)	62 (9.8)	631 (100.0)	37 (5.9)	25 (4.0)	62 (9.8)	*p* < 0.01
Follow-up study	415 (65.8)	216 (34.2)	631 (100.0)	86 (13.6)	130 (20.6)	216 (34.2)	*p* < 0.01

*Non-PEX* pseudoexfoliation syndrome not diagnosed; *PEX* pseudoexfoliation syndrome

During 10 years of follow-up (2016–2017) PEX prevalence increased to 34.2% (216 subject, (85 (39.4%) male and 131 (60.6%) female) (Table 1). There was no statistically significant difference of PEX frequency between males and females, 35.6 and 33.4%, respectively (*p* > 0.05). Mean age was significantly higher in the PEX group compared to the non-PEX group (73.01 ± 7.97 years vs 68.70 ± 8.16 years, *p* = 0.001).

The subjects’ distribution by sex and PEX occurrence is shown in Table 1.

PEX affected males and females in all age groups equally, while there was an increasing trend for the 10-year age-brackets: 23.3% of participants aged 55–65 years, 32.1% of those aged 66–75 years, and 47.3% of those aged 76–83 years were affected by PEX, this trend was statistically significant (*p* < 0.001) (Table 3). Comparing age group 55–65 and 66–75, 55–65 and 76–83, 66–75 and 76–83, significance was reached (*p* < 0.05; Table 3).

**Table 3:** The distribution of age groups by pseudoexfoliation syndrome in follow-up study

Age	Non-PEX, *n* (%)	PEX, *n* (%)	*p*-value
55–65^1^	158 (76.7)	48 (23.3)	0.001
66–75^2^	148 (67.9)	70 (32.1)	
76–83^3^	109 (52.7)	98 (47.3)	
Mean (± SD)	68.70 (8.16)	73.01 (7.97)	0.001
Median	68.0	74.0	

^1^´^2^´^3^Age groups compared: 55–65 and 66–75; 55–65 and 76–83; 66–75 and 76–83

*Non-PEX* pseudoexfoliation syndrome not diagnosed; *PEX* pseudoexfoliation syndrome

Those with PEX were statistically older in the follow-up study, age median in the PEX group was higher than that in the non-PEX group (74 vs 68 years respectively; *p* < 0.001; Table 3), and in the baseline study age medians in the PEX and non-PEX groups were 68 years versus 59 years (*p* < 0.001).

At baseline, there were 37 unilateral and 25 bilateral PEX cases, increasing to 86 unilateral (13.6%) and 130 bilateral (20.6%) cases in the 10-year follow-up (Tables 1 and 2). Of those with unilateral PEX at baseline, 20 became bilateral and nineteen (48.7%) remained unilateral during the 10-year follow-up (*p* < 0.001). A total of 154 (71.3%) new PEX cases were diagnosed at the 10-year follow-up (Table 2).

PEX risk factors of lifestyle are presented in Table 4. There was no statistically significant difference between those affected by PEX and those who were not PEX-subjects, in univariate logistic regression analysis.

**Table 4:** Distribution of life style and sociodemographic factors in pseudoexfoliation syndrome and pseudoexfoliation syndrome not diagnosed groups

Risk factors		Non-PEX, *n* (%)	PEX, *n* (%)	*p*-value
Smoking	Never	285 (68.7)	161 (74.5)	0.128
	Former	86 (20.7)	42 (19.4)	
	Current	44 (10.6)	13 (6.1)	
Alcohol	< 1time/month, never	274 (66.0)	145 (67.1)	0.742
	1–4 time/month	120 (28.9)	63 (29.2)	
	> 1 time/week	21 (5.1)	8 (3.7)	
BMI (kg/m^2^)	< 25.0	78 (19.2)	33 (15.6)	0.502
	25.0–29.99	150 (36.9)	79 (37.3)	
	≥ 30	178 (43.8)	100 (47.2)	
Education	Primary	32 (7.7)	24 (11.1)	0.334
	Secondary	143 (34.5)	75 (34.7)	
	University	240 (57.8)	117 (54.2)	
Marital status	Single	150 (36.1)	85 (39.4)	0.429
	Married	265 (63.9)	131 (60.6)	

*BMI* body mass index; *Non-PEX* pseudoexfoliation syndrome not diagnosed; *PEX* pseudoexfoliation syndrome

Age, gender, alcohol consumption, BMI, education and marital status did not increase the probability of having PEX.

In multivariate logistic regressions only age increased the risk of having PEX significantly (*p* < 0.001). Secondary education, in comparison with primary education showed a tendency for higher risk of having PEX in men (*p* = 0.08), but did not reach statistical significance.

Adjusting by multivariate risk factors, alcohol consumption 1–4 time/month and alcohol consumption > 1 time/week, marital status—married, former and current smoking, and normal weight reduced the probability of developing PEX in men, but not significantly.

Similarly, secondary and university education, normal weight and overweight reduced the probability of having PEX in women, but not significantly.

### Cataract

Of the available 1262 eyes, some degree of lens opacities was diagnosed in 1116 (95.1%) eyes and no cataract—in 58 (4.9%) eyes. Prevalence of cataract was significantly higher in the oldest age group, there was a 43.4% (95% CI 5.939–316.572, *p* < 0.001) increase in those in the oldest compared to the youngest groups. Comparing the youngest and middle age group, the risk to have cataract significantly increased 3.4 times (95% CI 1.833–6.330; *p* < 0.001). Cataract was diagnosed more frequently in females (63.3%) than in males (36.7%) (*p* = 0.01). There were statistically significantly more cataract diagnosed in the non-PEX group when compared to the study subjects with PEX (73.4 vs 26.6%, *p* > 0.05). Nuclear cataract was the most common type (70.7%) with no statistically significant difference between PEX/non-PEX groups. Mixed cataract was found frequently both in the non-PEX and the PEX groups (27.4 vs 28.9%; *p* > 0.05) (Table 5). We noticed a tendency in the PEX group to have a bigger risk of developing cataract by 21% (95% CI 0.576–2.574, *p* = 0.61); however, this trend was not statistically significant.

**Table 5:** Cataract distribution in pseudoexfoliation syndrome and pseudoexfoliation syndrome not diagnosed groups in follow-up study*

	Non-PEX, *n* (%)	PEX, *n* (%)	Total, *n* (%)	*p*-value
Cataract	819 (73.4)	297 (26.6)	1116 (100)	*p* > 0.05
Cataract forms				
Nuclear	591 (72.2)	198 (66.7)	789 (70.7)	*p* > 0.05
Cortical	2 (0.2)	0	2 (0.2)	
Subcapsular	2 (0.2)	0	2 (0.2)	
Mixed	224 (27.4)	99 (33.3)	323 (28.9)	

*Each eye was kept as object

*Non-PEX* pseudoexfoliation syndrome not diagnosed; *PEX* pseudoexfoliation syndrome

#### Age-related macular degeneration

Of the available 1197 eyes with comparable fundus imaging, any AMD was diagnosed in 937 (78.3%) eyes and no AMD in 260 (21.7%), respectively (Table 6). In the 10-year follow-up study, there were 383 new any AMD cases. There was no statistically significant difference in the prevalence of AMD between females and males (598 (63.8%) and 339 (36.2%), respectively, *p* = 0.18). The age-groups we had showed no statistically significant difference in the presence of any AMD nor was any association between PEX and AMD distribution (Table 6).

**Table 6:** Age-related macular degeneration distribution in pseudoexfoliation syndrome and pseudoexfoliation syndrome not diagnosed groups in follow-up study*

	Non-PEX	PEX	Total, *n* (%)	*p*-value
No AMD,* n* (%)	189 (21.1)	71 (23.7)	260 (21.7)	*p* > 0.35
AMD,* n* (%)	708 (78.9)	229 (76.3)	937 (78.3)	
Total, * n* (%)	897 (100.0)	300 (100.0)	1.197 (100.0)	

*Each eye was kept as object

*AMD* Age-related macular degeneration; *Non-PEX* pseudoexfoliation syndrome not diagnosed; *PEX* pseudoexfoliation syndrome

#### Central corneal thickness

CCT was measured in 1262 eyes of 631 patients. In the follow-up study, CCT was significantly thinner in the PEX group than in those without (529 ± 34 μm vs 532 ± 33 μm, respectively, *p* < 0.001). CCT became significantly thinner with age (*p* < 0.001), as it was 537 ± 30 μm in 55–65 years age group, 533 ± 33 μm in those aged 66–75 years, and 525 ± 36 μm in the over 75 s (*p* < 0.001). The thinnest central cornea was in the oldest age group both in the PEX and non-PEX groups (521 ± 33 μm; *p* < 0.001 vs 527 ± 37 μm; *p* = 0.006, respectively). Pearson correlation between age and CCT was weak but significant (*r* = −0,159; *p* < 0.001). There was no statistically significant difference between the PEX and non-PEX groups with respect to gender and CCT.

Over the 10 years of follow-up, CCT became statistically significantly thinner compared to baseline (baseline 539 ± 36 μm vs follow-up 531 ± 33 μm, *p* < 0.001. This also proved to be true in the non-PEX group (in baseline 539 ± 35 μm vs in follow-up 530 ± 31 μm, *p* = 0.002, respectively), but we were not able to confirm statistical significance in the PEX group (in baseline 539 ± 39 μm vs in follow-up 532 ± 36 μm, *p* = 0.280).

#### Corneal curvature

Of the 1182 eyes with corneal curvature (CC) measured, the mean CC was 7.7 ± 0.26 mm (in baseline mean CC 7.6 ± 0.27 mm). CC radius in males was statistically significantly higher than in females (7.8 vs 7.6 mm; *p* < 0.001). At follow-up, there was a tendency for CC to decrease with age, but not significantly (*p* > 0.05). At the 10-year follow-up, the cornea became statistically significantly flatter compared to baseline (7.6 ± 0.27 vs 7.7 ± 0.26 mm; *p* < 0.001), but there was no statistically significant difference between the PEX and non-PEX groups (PEX 7.64 ± 0.24 vs 7.68 ± 0.26, non-PEX 7.66 ± 0.28 vs 7.70 ± 0.27 mm; *p* = 0.123).

#### Corneal astigmatism

Of the 1182 eyes with relevant measurements, 290 cases of corneal astigmatism were found. Females had more corneal astigmatism (62.8 vs 37.2%, *p* = 0.02). In the study population, corneal astigmatism was mostly with-the-rule, and this tendency was evident in both PEX and non-PEX groups other than in the oldest group where against-the-rule corneal astigmatism was statistically significantly more common (*p* < 0.001). In the baseline study, this group displayed the same trend, but it was then not statistically significant.

## Discussion

PEX typically affects the anterior segment of the eye and its worldwide prevalence is estimated to be 0.2–30.0% of people older than 60 years of age[5, 6, 12, 22–26]. Consistent evidence of PEX incidence and prevalence is lacking [3] and previous studies differed based on geographical, ethnic and racial features, as well as in age and gender distributions and diagnostic methods, making comparison difficult.

In Lithuania, PEX prevalence was found to be 47.3% in 76–83-year-old age group, which is slightly higher than a population with similar characteristics in Iceland (40.6% of those who are 80 years old) [6]. PEX prevalence increased with age in all studies [9, 12, 13, 23, 26–29] and this is in agreement with our finding, as in our study PEX subjects were older than those without it (72.2 ± 8.1 years vs 68.6 ± 8.2 years, respectively). Similarly to our results, in Turkey the highest PEX rate was in the 80 years old patients (18.4%) with an increased odds ratio of 45.78 (p < 0.01) when compared to the 40–49 age-group [23].

A previous Lithuanian study [17] found that PEX risk increased by 13.5% with each additional year of life (95% CI 1.1–1.17; *p* < 0.001), while a Swedish study established the annual PEX incidence as 1.8% (95% CI 1.3–2.4) [7]. The Reykjavik Eye Study found that on average there was a 5% increase in the risk of developing definite PEX for every decade of life in people older than 50 years of age (OR = 1.05; 95% CI 1.01–1.09, *p* = 0.022) [10].

Incidence data have been provided by a few population based studies such as the Reykjavik Eye Study (5- and 12-year follow-up) [9, 30], the study by Aström et al. [7] in Skelleftea, Northern Sweden (21-year follow-up), Chennai Eye Disease Incidence Study with 6-year follow-up [31]. In the Thessaloniki study the 12-year incidence of PEX was 19.6% (95% CI, 17.1-22.2) [32], similar to ours.

Lithuania is situated on the eastern shore of the Baltic Sea in Northern Europe. In our study, PEX prevalence in Lithuania was found to be 34.2%, higher than previously noted in the neighboring Nordic countries (Sweden—23%, Finland—22%, Iceland—10,7%) [6, 7, 33]. In the 76–83-year-old age group, Lithuanian subjects had similar prevalence to that of other Scandinavian countries: 47.3% in our study that is only slightly higher than in Iceland (40.6% of those who are 80 years old) [6].

In other Baltic states, Estonia recorded 35.4% prevalence in 2004 [11] and 25.5% in 2010 [34], while Latvia reported lower prevalence of 21.6% [35]. Our estimates compare well to similar background of the other Baltic states.

The age-related PEX progression from unilateral to bilateral disease is well established [9]. In our study 53.0% of unilateral PEX progressed to bilateral PEX in 10-year period, this is very similar to the progression rate seen both in Iceland and Sweden [7, 9].

In contrast to some other studies we did not find significant gender differences in our cohort, although there are studies that strengthen our observations as well [26, 36, 37]. Clearly, larger and well characterized samples with detailed ophthalmic examination are required to enable better understanding of this issue.

Not many studies have been able to analyze lifestyle and other factors. In a Saudi Arabian study no significant relation was found between education level, occupation and lifestyle of the patients and the prevalence of PEX [37]. While our study showed some trends in lifestyle and demographic characteristics, none were significant, this is in agreement with the findings of the Thessaloniki Eye Study [32]. It might be worthwhile though pursuing these further if a suitably large cohort is established.

Reykjavik Eye Study had additional data analyzed for PEX risk factors older age, female sex, increased iris pigmentation, moderate use of alcohol, and asthma were all associated with higher PEX prevalence, whereas the consumption of vegetables and fruit was associated with lower PEX prevalence [38]. Five years later, analyzing the same risk factors, significant associations were found only with age and the consumption of fruit [38], but by 12 years there were no statistically significant associations [9, 10, 32]. Being married was shown to be a protective factor for definite PEX in a univariate analysis, but after stratification by the effect of age, the association disappeared [9, 30].

Chennai Eye Disease Incidence Study showed a significant relationship between older age, illiteracy, rural residence, pseudophakia and nuclear cataract and the 6-year incidence of PEX [32, 39]. In our study secondary education for men showed a tendency higher risk of having PEX. Controlling for risk factors (former and current smoking, and normal weight) in a multivariate logistic regression showed the tendency to reduce the probability of having the PEX.

Radius of CC was found to be age-independent and significantly steeper in females than in males [40] and our findings agreed with these. In our study the cornea became flatter during the 10-year follow-up (7.6 vs 7.7 mm; *p*<0.001). And Hepsen et al. reported significantly steeper corneal curvature in PEX eyes compared to those without PEX [41] but we were unable to confirm this in our study [42].

In Reykjavik Eye Study CCT was found to be independent of age and gender [40]: While our findings regarding gender were the same, we did notice that CCT became significantly thinner with aging (*p*<0.001). Some studies claimed that in PEX, CCT values were significantly lower than those in non-PEX eyes [43, 44] and our findings are in agreement with these. However, Krysik, for instance, found CCT to be thicker in the PEX group [45]. Many other authors reported that there was no significant difference in CCT between the PEX and non-PEX groups [10, 41, 42, 46–48]. Again, a large enough cohort might provide an answer to these conflicting data.

Lens opacification occurs in a high proportion of PEX eyes, resulting in cataract surgeries [34] most commonly nuclear [49]. Nuclear sclerosis was predominant in PEX eyes, compared to those without PEX (57.6 and 36.9%, respectively) [11], confirmed by Blue Mountains Eye Study [50]. In contrast, while we found a large number of nuclear cataract both in the PEX and non-PEX groups, we could not confirm significant difference in our population. In contrast, Gunes found that mixed cataract was the most common cataract type in the PEX patients in Turkey [51]. In a 30-year follow-up study Ekström et al. found that PEX was the second most important predictor for cataract surgery after lens opacities, accounting for a 2.38-fold increased risk with man having a lower risk for cataract formation [52].

In a Greek study the presence of AMD was strongly related to PEX (*x*^2^ = 13.675, *p* = 0.003; Mantel-Haenszel test=13.66, *p* = 0.002) [53], while in a Turkish population, the prevalence of AMD was found to be significantly higher in the PEX group than in the non-PEX (17.9 vs 9.5%, *p* = 0.03) [51], but in our study we could not confirm these findings.

### Strength and limitations

The main strength of this study is its population-based prospective study design. Detailed ophthalmological examinations were carried out by trained and certified operators using a strict protocol.

The main limitation includes response rate of 66.4%, limiting the generalizability of the study. Cataract surgery had already been performed on 8.02% of those returning for 10-year follow-up, unfortunately there was no information on their PEX status and this might have impacted on our incidence rate [54].

## Conclusion

PEX prevalence in this Lithuanian cohort of well characterized patients increased from 9.8 to 34.2% with no significant difference between sexes, but with significant increase with age. Our results showed that detailed characterization of the PEX and non-PEX eyes was beneficial in identifying trends in anterior segment changes. Conversely, there was no significant relationship with any AMD findings for these patients. An appropriate clinical pathway is required to enable timely diagnosis and treatment of these patients in order to keep them active in society.

## Supplementary Information

Below is the link to the electronic supplementary material.

Supplementary file1 (PDF 475 kb)

## Data Availability

The data used to support the findings of this study are available from the corresponding author upon request. PEX associations with cardiovascular diseases were discussed in our earlier article [54] (PMID: 31956932; 10.1007/s10792-019-01262-x; https://www.ncbi.nlm.nih.gov/pubmed/?term=rumelaitiene).
