# Supplementary material for: Exploring association between pseudoexfoliation syndrome and ocular aging
Source: Int Ophthalmol. 2022 Sep 21;43(3):847–57. doi: 10.1007/s10792-022-02486-0 (PMC10042963; doi:10.1007/s10792-022-02486-0)
Supplement: Supplementary file 1 — Supplementary file1 (PDF 475 kb) [file 10792_2022_2486_MOESM1_ESM.pdf]

Klausimyno pildymo data

Metai	Mėnuo	Diena

Sveikas senėjimas

Asmens klausimynas

Vardas: .... Pavardė: ....

Gimimo data

Metai	Mėnuo	Diena

Tiriamojo identifikacijos numeris

☐ ☐ ☐ ☐ ☐ ☐ ☐

Žmogaus, imančio interviu, kodas

G. ROSE KLAUSIMYNAS

Prašome atsakyti į žemiau pateiktus klausimus. Reikiamą atsakymą pažymėkite langelyje X ženklu.

1. Ar Jūs kada nors jautėte skausmą ar nemalonius pojūčius krūtinėje?

☐ 1. Taip
☐ 2. Ne

1a. Ar Jūs kada nors jautėte spaudimą ar sunkumą krūtinėje?

☐ 1. Taip
☐ 2. Ne

*Jei **NE**, prašau pereiti prie **8-to klausimo,** jei **TAIP** – klausinėkite toliau.*
*Jei atsakymas sutampa su pažymėtuoju *, pereikite prie 8-to klausimo.*

2. Ar jis atsiranda, kai Jūs einate į kalną, skubate?

☐ 1. Taip
☐ 2.* Ne*
☐ 3. Niekada neskubu ir nelipu į kalną

3. Ar jis atsiranda, kai Jūs įprastu žingsniu einate lygia vieta?

☐ 1. Taip
☐ 2. Ne

4. Kaip Jūs elgiatės, jei skausmas Jums atsiranda einant?

☐ 1. Sustoju ar sulėtinu žingsnį
☐ 2.* Nemažindamas tempo einu toliau*
☐ 3. Išgeriu nitroglicerino tabletę

Jei asmuo pasipurškia ar po liežuviu padeda Nitrogliceriną ir eina toliau, pažymėkite atsakymą „sustoju ar sulėtinu žingsnį“.

5. Jei Jūs ramiai stovite, kaip keičiasi skausmas?

☐ 1. Sumažėja
☐ 2.* Nesumažėja*

6. Per kiek laiko sumažėja skausmas?

☐ 1. Per 10 minučių ir greičiau
☐ 2.* Daugiau kaip per 10 minučių*

7. Galite patikslinti, kurioje vietoje atsiranda skausmas ar nemalonūs pojūčiai? (prašau pasirinkti visas tinkamas vietas)

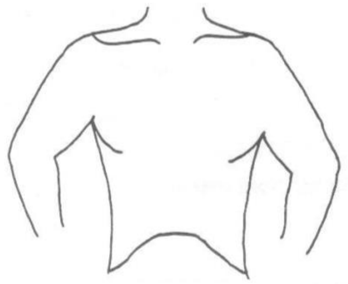

- ☐ 1. Krūtinkaulis (viršus arba vidurys)
- ☐ 2. Krūtinkaulis (apačia)
- ☐ 3. Priekinė kairė krūtinės pusė
- ☐ 4. Kairė ranka
- ☐ 5. Kita Prašau patikslinkite: ______________

8. Ar gydytojas Jums kada nors sakė, jog sergate STENOKARDIJA (KRŪTINĖS ANGINA)?

- ☐ 1. Taip
- ☐ 2. Ne

Jei Ne, pereiti prie 9-to klausimo

Jei **Taip**, kada ji buvo diagnozuota pirmą kartą?

**Metai**	**Mėnuo**

9. Ar Jums kada nors buvo kuris nors iš šių? Jei **Taip**, prašau pateikite metus, mėnesį ir ligoninę.

	**Taip**	**Ne**	**Metai**	**Mėnuo**	**Ligoninė / klinika**
**Angioplastika, vainikinių arterijų gydymas, praplečiant balionėliu**					
**Vainikinių kraujagyslių nuosrūvio operacija (šuntavimas) (CABG)**					

BENDRASIS KLAUSIMYNAS

Prašome atsakyti į žemiau pateiktus klausimus. Reikiamą atsakymą pažymėkite langelyje X ženklu.

1. Koks yra Jūsų išsilavinimas?

☐ 1. Nebaigtas pradinis arba nėra jokio oficialaus išsilavinimo
☐ 2. Pradinis
☐ 3. Profesinis (mokymasis amato)
☐ 4. Vidurinis
☐ 5. Aukštesnysis (technikumas)
☐ 6. Universitetinis (laipsnis)

2. Kokia Jūsų šeiminė padėtis?

☐ 1. Viengungis (netekėjusi)
☐ 2. Vedęs (ištekėjusi)
☐ 3. Gyvena kartu poroje
☐ 4. Išsituokęs (-usi)
☐ 5. Našlys (-ė)

Prašau pasakykite, ar kuri nors šių ligų kada nors Jums buvo diagnozuota?

3. Ar gydytojas Jums kada nors sakė, kad Jūs sirgote MIOKARDO INFARKTU?

☐ 1. Taip
☐ 2. Ne

Jeigu TAIP, kokiais metais ir kokioje ligoninėje buvote gydomas/-a?

				Kalendoriniai metai

4. Ar gydytojas Jums kada nors sakė, kad Jūs sirgote INSULTU?

☐ 1. Taip
☐ 2. Ne

Jeigu TAIP, kokiais metais ir kokioje ligoninėje buvote gydomas/-a?

				Kalendoriniai metai

5. Ar gydytojas yra kada nors Jums sakęs, kad Jūsų **kraujospūdis padidėjęs**?

☐ 1. Taip
☐ 2. Ne

Jei **TAIP**, ar Jūs pastarąsias dvi savaites vartojote vaistus dėl padidėjusio kraujospūdžio?

☐ 1. Taip
☐ 2. Ne
☐ 3. Nežinau

6. Ar gydytojas yra kada nors Jums sakęs, kad Jūs sergate **cukralige**?

☐ 1. Taip
☐ 2. Ne

Jei **TAIP**, kaip Jūs esate gydomas/-a?

☐ 1. Tik dieta
☐ 2. Dieta ir insulinu
☐ 3. Dieta ir tabletėmis
☐ 4. Dieta, tabletėmis ir insulinu
☐ 5. Nėra gydymo (neskirtas gydytojo)

7. Ar gydytojas yra kada nors Jums sakęs, kad Jums padidėjęs **cholesterolio kiekis kraujyje**?

☐ 1. Taip   Jei **TAIP**, kaip Jūs esate gydomas/-a?
☐ 2. Ne

☐ 1. Tik dieta
☐ 2. Dieta ir tabletėmis
☐ 3. Tik tabletėmis
☐ 4. Nėra gydymo (neskirtas gydytojo)

8. Kiek valandų įprastai per tipišką savaitę Jūs užsiimate daug fizinių pastangų reikalaujančia veikla, tokia kaip namų ruoša, darbas sode, namo priežiūra, išskyrus, kai dirbate darbe? ☐☐☐

9. Kiek valandų įprastai per tipišką savaitę Jūs sportuojate, žaidžiate judrius žaidimus, vaikštote pėsčiomis? ☐☐☐

10. Ar Jūs rūkote cigaretes?

☐ 1. Taip, reguliariai, vidutiniškai mažiausiai vieną cigaretę per dieną
☐ 2. Taip, retkarčiais, mažiau nei vieną cigaretę per dieną
☐ 3. Ne, rūkiau anksčiau, bet mečiau
☐ 4. Ne, niekada nerūkiau

Esamiems ir buvusiems rūkaliams: kiek cigarečių per dieną surūkote šiuo metu? (arba surūkydavote, jei metėte)? ☐☐☐

Esamiems ir buvusiems rūkaliams: kokio amžiaus buvote, kai pradėjote rūkyti? ☐☐ metų

Buvusiems rūkaliams: kokio amžiaus buvote, kai metėte rūkyti? ☐☐ metų

Buvusiems rūkaliams: kada Jūs metėte rūkyti? ☐☐☐☐ Kalendoriniai metai

11. Kaip dažnai per praėjusiuosius 12 mėnesių Jūs vartojote alkoholį?

☐ 1. Kiekvieną dieną arba beveik kiekvieną dieną
☐ 2. Apie 2-4 kartus per savaitę
☐ 3. Apie kartą per savaitę
☐ 4. Apie 1-3 kartus per mėnesį
☐ 5. Mažiau kaip kartą per mėnesį
☐ 6. Niekada per paskutiniuosius metus

12. Kiek alaus (litrais) Jūs paprastai išgeriate vienu metu? ☐☐ , ☐

13. Kiek vyno (ml) Jūs paprastai išgeriate vienu metu? ☐☐☐

14. Kiek stipriųjų gėrimų (ml) Jūs paprastai išgeriate vienu metu? ☐☐☐

15. Kokį kiekį alkoholio išgėrėte per praėjusią savaitę (7 dienas)?

a) alaus (litrais) ☐☐ , ☐

b) vyno ar šampano (ml) ☐☐☐☐

c) degtinės, konjako ar kitų stiprių gėrimų (ml) ☐☐☐☐

Dabar noriu paklausti keletą klausimų apie Jūsų mitybą.

16. Kokius riebalus Jūs dažniausiai vartojate ruošdami maistą?

- ☐ 1. Dažniausiai aliejų
- ☐ 2. Dažniausiai margariną
- ☐ 3. Dažniausiai sviestą
- ☐ 4. Dažniausiai taukus
- ☐ 5. Dažniausiai maišau aliejų su kitais riebalais
- ☐ 6. Nevartoju jokių riebalų
- ☐ 7. Maisto namuose negaminu

17. Kokius riebalus Jūs dažniausiai vartojate sutepti sumuštiniams?

- ☐ 1. Jokių
- ☐ 2. Liesą margariną (40-60% rieb. –„Voimix“, „Rama“, „Boni“, „Delma“, „Vilnius“ ir kt.)
- ☐ 3. Riebų margariną (80% rieb. –„Kronella“, „Hushals“ ir kt.)
- ☐ 4. Margariną, bet nežinau riebumo
- ☐ 5. Sviestą, tepųjį mišinį
- ☐ 6. Taukus
- ☐ 7. Kita

18. Kiek jų tepate ant riekės?

- ☐ 1. Netepu
- ☐ 2. Labai mažai (persišviečia)
- ☐ 3. Vidutiniškai (nepersišviečia)
- ☐ 4. Daug (storai)

19. Kiek stiklinių pieno arba (ir) kefyro (rūgštaus pieno, jogurto) paprastai išgeriate per savaitę?

1. Pieno		
2. Kefyro		
3. Rūgštaus pieno (rūgpienio)		
4. Jogurto		

20. Kokį pieną dažniausiai geriate?

- ☐ 1. Nenugriebtą (natūralų) karvės pieną
- ☐ 2. Standartinį nepagerintą (3,2-2,5% rieb.)
- ☐ 3. Liesą (0,5-1,0% rieb.)
- ☐ 4. Negeriu pieno

21. Kiek kiaušinių (virtų ar keptų) Jūs paprastai suvalgote per savaitę? ☐☐

22. Kaip dažnai Jūs valgote šiuos maisto produktus?

**Maisto produktai**	**Kasdien**	**4-6 k. per sav.**	**2-3 k. per sav.**	**Kartą per sav.**	**Kartą ar kelis k. per mėn.**	**Rečiau ar niekada**
Bulves						
Košes ar dribsnius						
Fermentinį sūrį (geltoną)						
Varškės sūrį (baltą)						
Vištieną						
Žuvį						
Mėsą						
Dešras						
Šviežias morkas: a) vasarą ir rudenį						
b) žiemą ir pavasarį						
Kitas šviežias daržoves: a) vasarą ir rudenį						
b) žiemą ir pavasarį						
Virtas daržoves						
Šviežius vaisius ar uogas: a) vasarą ir rudenį						
b) žiemą ir pavasarį						
Natūralias sultis: a) vasarą ir rudenį						
b) žiemą ir pavasarį						
Saldumynus: a) saldainius, šokoladą						
b) pyragaičius, tortą						

DĖKOJAME, KAD UŽPILDĖTE KLAUSIMYNĄ

Kartas nuo karto mes norėtume susisiekti su Jumis ir užduoti keletą trumpų klausimų apie Jūsų sveikatą. Jei sutinkate, užrašykite savo telefono numerį.

Telefono numeris

KRAUJOTAKOS SISTEMOS VERTINIMAS

**Arterinis kraujo spaudimas:**	**I**	**II**	**III**
Sistolinis (mmHg)	□□□	□□□	□□□
Diastolinis (mmHg)	□□□	□□□	□□□
Pulsas (k/min.)	□□□	□□□	□□□

Antropometriniai matavimai:

Ūgis (stovint be batų) (cm) □□□,□

Svoris (be batų. apatiniais baltiniais) (kg) □□□,□

Liemens apimtis (cm) □□□,□

Klubų apimtis (cm) □□□,□

ELEKTROKARDIOGRAMA Nr. □□□

EKG neužrašyta-1 □
Techniškai nepilnavertė EKG-2

Q ir QS 1 □□

QRS ašies nukrypimas 2 □

Aukšta R dantelio amplitudė 3 □

S-T (J) nusileidimas 4 □□

T dantelio pakitimai 5 □

A-V laidumo sutrikimai 6 □□

Intraskilvelinio laidumo sutrikimai 7 □□

Aritmijos 8 □□

8 □□

Mišrūs pakitimai 9 □

9 □

9 □□

Pulso dažnumas pagal EKG, k/min. □□□

Tyrėjo kodas .... □

OFTALMOLOGINIO IŠTYRIMO ANKETA

1. Lytis: vyras – **1**, moteris – **2** ☐
2. Nusiskundimai:
3. Anamnezė: akių ligos nenustatytos – **1**, AM – **2**, glaukoma – **3**, katarakta – **4**, DR – **5**, trauma – **6**, tinklainės atšoka – **7**, aukšto laipsnio trumparegystė – **8** ☐☐☐ OD ☐☐☐ OS
4. Vartojami vaistai akių ligoms gydyti
5. Vartojami vaistai bendrinėms ligoms gydyti
6. Optinės korekcijos priemonė: netaiko – **1**, akiniai – **2**, kontaktiniai lęšiai - **3** ☐

	OD	OS
7. Buvusios operacijos		
8. Anoftalmus	☐	☐
9. Autorefraktometrija	sph cyl ax	sph cyl ax
10. Keratometrija (D)	K1☐☐,☐☐ ☐☐☐	K1☐☐,☐☐ ☐☐☐
	K2☐☐,☐☐ ☐☐☐	K2☐☐,☐☐ ☐☐☐
11. Regos aštrumas (LogMAR)		
be korekcijos	☐,☐☐	☐,☐☐
korekcija	sph cyl ax	sph cyl ax
su korekcija	☐,☐☐	☐,☐☐
12. Ragena: be pakitimų – **1**, drumstys – **2**, degeneraciniai pokyčiai – **3**	☐	☐
13. Rainelių spalva: šviesi – **1**, tamsi – **2**	☐	☐
Sol. Proparacaini 0,5%		
14. Centrinis ragenos storis (µm) 1)	☐☐☐	☐☐☐
2)	☐☐☐	☐☐☐
3)	☐☐☐	☐☐☐
15. Tn (mmHg) 1)	☐☐,☐	☐☐,☐
2)	☐☐,☐	☐☐,☐
3)	☐☐,☐	☐☐,☐
Sol. Cyclopentolati 1%		
16. Lęšiuko įvertinimas pagal LOCS III NO	☐,☐	☐,☐
NC	☐,☐	☐,☐
C	☐,☐	☐,☐
P	☐,☐	☐,☐

17. Pseudoeksfoliacijos: nėra – **1**, lęšiuko kaps. centre – **2**, lęšiuko kaps. periferijoje – **3**, rainelės vyzdiniame krašte – **4**, ragenos užp. paviršiuje – **5** ☐ ☐

	OD	OS
18. Vyzdžio skersmuo	☐☐,☐	☐☐,☐
19. Priekinio segmento nuotraukos nr.	☐☐☐☐	☐☐☐☐
20. Dugno nuotraukos nr. ☐☐☐☐ 60˚	☐☐☐☐-☐☐☐☐	☐☐☐☐-☐☐☐☐
21. OKT tyrimas: makulos storis (µm)	☐☐☐	☐☐☐

22. Makulos kontūras
23. Kiti pakitimai
24. Akipločio tyrimas: MD
25. PSD
26. Apibūdinimas
27. Pradėta Baigta
28. Pastabos
